# Voltage-induced penetration effect in liquid metals at room temperature

**DOI:** 10.1093/nsr/nwz168

**Published:** 2019-11-05

**Authors:** Frank F Yun, Zhenwei Yu, Yahua He, Lei Jiang, Zhao Wang, Haoshuang Gu, Xiaolin Wang

**Affiliations:** 1 Institute for Superconducting and Electronic Materials, Australian Institute for Innovative Materials, University of Wollongong, Wollongong, NSW 2500, Australia; 2 Laboratory of Bioinspired Smart Interfacial Science, Technical Institute of Physics and Chemistry, Chinese Academy of Sciences, Beijing 100190, China; 3 Hubei Key Laboratory of Ferro & Piezoelectric Materials and Devices, Faculty of Physics and Electronic Sciences, Hubei University, Wuhan 430062, China; 4 ARC Centre of Excellence in Future Low-Energy Electronics Technologies, University of Wollongong, Wollongong, NSW 2500, Australia

**Keywords:** penetration effects, liquid metals, surface tension, porous materials, superwet

## Abstract

Room-temperature liquid metal is discovered to be capable of penetrating through macro- and microporous materials by applying a voltage. The liquid metal penetration effects are demonstrated in various porous materials such as tissue paper, thick and fine sponges, fabrics, and meshes. The underlying mechanism is that the high surface tension of liquid metal can be significantly reduced to near-zero due to the voltage-induced oxidation of the liquid metal surface in a solution. It is the extremely low surface tension and gravity that cause the liquid metal to superwet the solid surface, leading to the penetration phenomena. These findings offer new opportunities for novel microfluidic applications and could promote further discovery of more exotic fluid states of liquid metals.

## INTRODUCTION

Penetration through a solid with micro- or nanopores is one of the three fascinating macroscopic phenomena that are well known in superfluids such as liquid helium [1–4]. It is the zero viscosity that endows the liquid helium superfluid with the ability to flow without any resistance, leading to its amazing penetration and other superfluid effects. Nevertheless, the helium superfluid penetration effect emerges only in the so-called quantum states or quantum fluids at extremely low temperatures of almost zero Kelvin. In contrast, conventional liquids such as water and oils can diffuse into or penetrate through a solid with macropores at room temperature as a result of the capillary effect, although their surface tensions are not low enough to enable them to penetrate through porous materials. In this work, we propose to study, for the first time, the possible penetration effects of gallium-based liquid metals, since their surface tension can be significantly and easily tuned by the voltage at room temperature.

Recently, room-temperature liquid metals, such as liquid gallium and its eutectic alloys, have attracted great attention in many research fields due to their unique chemical and physical properties, such as negligible vapor pressure [5], large surface tension [6], low toxicity [7], and high electrical and thermal conductivity [5,8]. We highlight some typical phenomena observed in liquid gallium and its alloys under the application of a voltage at room temperature: (i) giant deformation in acid or base solutions [9–11]; (ii) self-rotation [12,13]; (iii) locomotion [13,14]; (iv) spontaneous fast deformation and solidification in the supercooled state [15]; (v) the electro-hydrodynamic shooting phenomenon [16]; (vi) non-contact and maskless electrochemical patterning or lithography [17]; (vii) the phagocytosis effect [18]; (viii) the triggered wire oscillation effect [19]; (ix) the liquid metal heartbeat effect [20]. Note that phenomena (iv), (vi), and (ix) were recently discovered by our group. It is noteworthy that the surface tension of liquid gallium and its alloys is quite high (∼500 mJ/m^2^) [5], and therefore does not wet most solids. When a layer of oxide, e.g. Ga_2_O_3_, is formed on the surface, however, due to the electrochemical reactions in acid or alkaline solutions, the surface tension is then reduced greatly and can reach a near-zero value under an applied voltage [9,21]. It is well accepted that the extremely low surface tension plays a key role in the giant deformation effect in liquid metals [10]. Furthermore, there have been numerous studies exploring the wettability properties and dynamic behaviors of liquid metals with different substrates [22–25]. The results indicate that both morphology and roughness of substrate would influence the surface energy and dynamic behaviors.

Inspired by the near-zero surface tension phenomenon, here, we propose to explore liquid metal’s new capability of penetrating through porous materials with the help of both voltage and gravity. In this work, we demonstrate the liquid metal penetration effect in various porous materials such as tissue paper, fine sponges, fabrics, and meshes.

## RESULTS AND DISCUSSION

**Figure 1.**
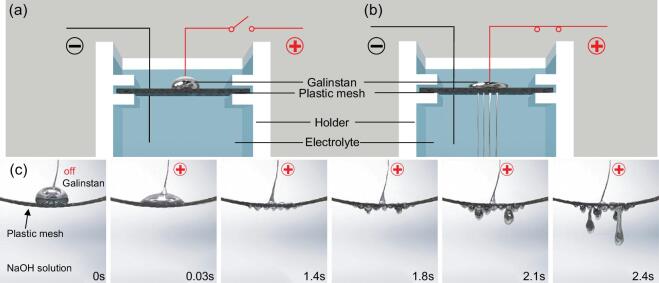
The penetration effect of galinstan through voltage control in an electrolyte. Schematic diagram of a galinstan droplet on a plastic mesh before (a) and after (b) the voltage is applied. (c) Snapshots of the penetration effect for a plastic mesh with 5 V applied voltage in 1 mol/L NaOH solution.

The experimental set-up for the penetration effect is shown in Fig. 1a and b. A galinstan droplet is placed on the top surface of a porous material that is fixed inside a PTFE-coated container. The whole set-up is immersed in NaOH electrolyte. A copper wire is inserted into the galinstan droplet to act as an anode, and another copper wire to act as a cathode is placed in the electrolyte. Different porous materials with various average pore sizes and thicknesses were used. All experiments were conducted using different voltages and solution concentrations at room temperature.

We first demonstrate our observations on the liquid metal penetration effect using a plastic mesh 0.2 mm in thickness and with 0.75 mm pore size. The galinstan droplet remains stationary and has a spheroidal shape. When a voltage is applied, the droplet deforms rapidly within 0.03 s and becomes totally flat at the time *t* = 1.4 s on the plastic mesh surface. When *t* > 1.8 s, it starts to penetrate through the pores of the plastic mesh, and then the droplet continues to flow down, forming thin threads and eventually touching the bottom of the container. Some of the threads are cut off before reaching the bottom, depending on the sizes of the droplets. Snapshots of the penetration effect for the plastic mesh are shown in Fig. 1c.

**Figure 2.**
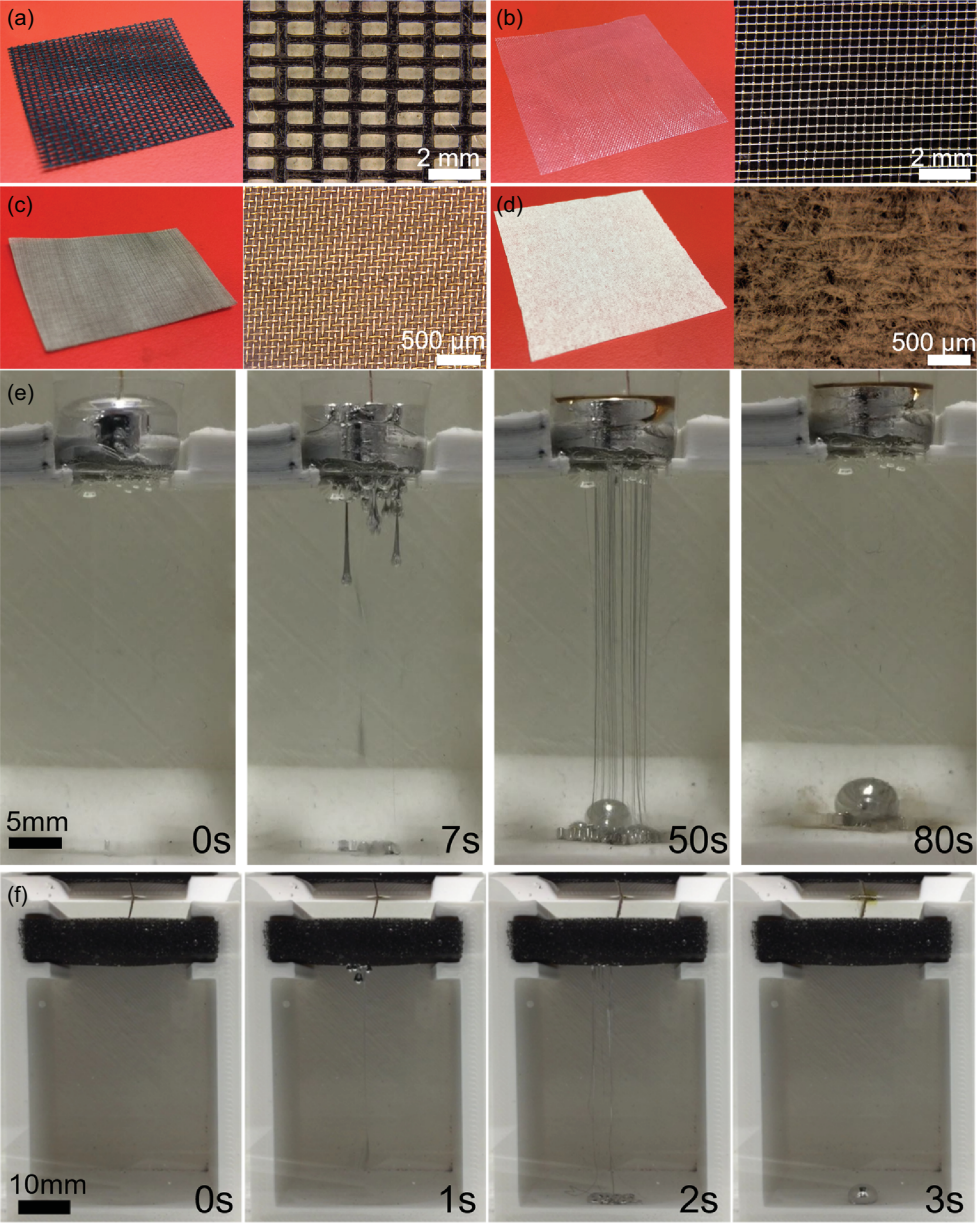
Photographs and optical microscope images of (a) plastic mesh, (b) fiber mesh, (c) metal mesh, and (d) tissue paper. Snapshots of the penetration effect for (e) metallic mesh with pore size of 45 μm under 5 V applied voltage in 1 mol/L NaOH solution and (f) 7.5 mm thick fine sponge with a 10 V applied voltage in 0.5 mol/L NaOH solution.

We have further verified the liquid metal penetration effect for other porous materials such as fabric meshes, metal meshes, and tissue paper with different pore sizes. These experiments were conducted under a voltage of 5 V, in a 1 mol/L NaOH solution with 0.2–1 mm in thickness (Fig. 2a–d). The galinstan droplets can penetrate through all selected porous materials and flow out from the other side. Snapshots of galinstan penetrating through a metallic mesh with a pore size of ∼45 μm are shown in Fig. 2e. A few typical snapshots showing the penetration effect for tissue paper (with an average pore size of a few microns), fabric (∼280 μm) and plastic (∼750 μm) meshes are provided in Fig. S1 in the supporting information. We can see that the penetration process takes place for all thin porous materials.

**Figure 3.**
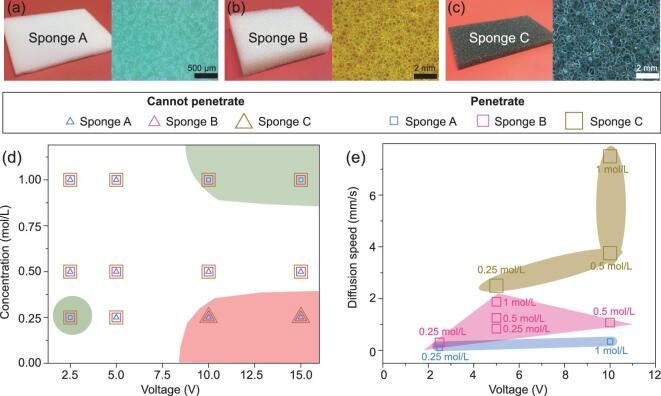
Experimental conditions for liquid metal penetration through sponges. Photographs and optical microscope images of (a) sponge A (melamine foam with pore size of ∼150 μm), (b) sponge B (polyurethane foam with pore size of ∼350 μm), and (c) sponge C (polyurethane foam with pore size of ∼550 μm). (d) Reference diagram of the liquid metal penetration effect under voltages of 2.5–15 V in NaOH concentrations of 0.25–1 mol/L. The square symbol or triangle symbol indicates that the liquid metal can or cannot penetrate through the sponges. The background of the reference diagram is colored green and red accordingly, to represent each region (where it can and cannot penetrate through all the sponges) discussed in the text. (e) Diffusion speeds of the liquid metal penetration effect for sponges A, B, and C under applied voltages of 2.5–10 V in NaOH concentrations of 0.25–1 mol/L.

It is also possible to demonstrate the liquid metal penetration phenomenon using thicker sponges with thicknesses of up to 10 mm and varying pore sizes (Video S1). Three sponges with average pore sizes of ∼150 (sponge A), ∼350 (sponge B), and ∼550 μm (sponge C) were used to again reveal the penetration effect. Our results show that the pore sizes are proportional to the speed at which the liquid metal penetrates through the sponges, under the same magnitude of applied DC voltage and the same solution concentration: a larger pore size corresponds to a faster flow through the thick sponges. Figure 2f shows snapshots of the penetration effect for a 7.5 mm thick sponge. Remarkably, upon application of a voltage, the droplet immediately merges into the sponge, and then quickly (*t* = 1 s) emerges from the other side of the sponge, and then continuously flows down to the bottom of the container. This process continues if the voltage is kept on, and it can be stopped quickly, either by turning off the applied voltage or exhausting the supply of the galinstan droplets.

To investigate the voltage and electrolyte concentration effects on the liquid metal penetration phenomenon, we employed DC voltages of 2.5 up to 15 V in NaOH solutions with a concentration of 0.25, 0.5, and 1 mol/L for sponges A, B, and C, as shown in Fig. 3a–c. The volume of galinstan used was fixed at 150 μL for all experiments. Figure 3d shows the experimental conditions, which help us to find out under what conditions the liquid metal can penetrate through a sponge, such as the average pore size of the sponge, the applied voltage, and the concentration of the electrolyte solution. Figure 3d contains regions with three typical sets of conditions. For green-region conditions, the liquid metals can penetrate through all the sponges. For high voltages and low concentrations (red region), however, no penetration is observed, which is likely due to the rapid reaction that produces excessive oxides on the surface of the droplet, which cannot be dissolved in the NaOH solution promptly. The excess of solid oxides on the outside restricts the flow of the inner liquid metal, making the liquid metal more solid-like. Furthermore, for conditions in the unshaded area, penetration can only take place for sponges B and C. Figure 3e shows the relationship between the diffusion speed of the liquid metal, the applied voltage, and electrolyte concentration. For sponge B, the speed increases from 0.8 to 1.9 mm/s as the NaOH electrolyte concentration increases from 0.25 to 1 mol/L with voltage values fixed at 5 V. Additionally, when voltage increases from 2.5 to 5 V with a fixed concentration of 0.25 mol/L, the diffusion speed raises from 0.3 to 0.8 mm/s. Thus, both the voltage and NaOH concentration can affect the penetration speed of liquid galinstan. Due to the formation of the oxide layer on galinstan under an applied voltage, this oxide layer would then be dissolved in NaOH solution. However, as long as the voltage is kept on, there is always an oxide layer formed on the liquid metal surface, which keeps the surface energy extremely small. This oxidation/dissolution dynamic process enables the penetration effect and determines the penetration speed, which can be controlled by adjusting the values of voltage and electrolyte concentration. According to our observations, the maximum volume of the galinstan that can penetrate through a sponge is unlimited under the appropriate applied voltage/electrolyte concentration corresponding to different porous materials, provided that the galinstan is supplied continuously. Moreover, we also found that the penetration speed increases with increasing pore size of the sponge for all conditions. As shown in Fig. 3e, the penetration speeds for sponges A, B and C are in the ranges of 0.1–0.34, 0.3–1.9 and 2.5–7.5 mm/s (Videos S2 –4), respectively. A few typical snapshots showing the penetration effect under different conditions for different sponges are given in Figs S2 and S3. We can conclude that the penetration process mainly depends on the oxidation/dissolution rate and the pore size.

**Figure 4.**
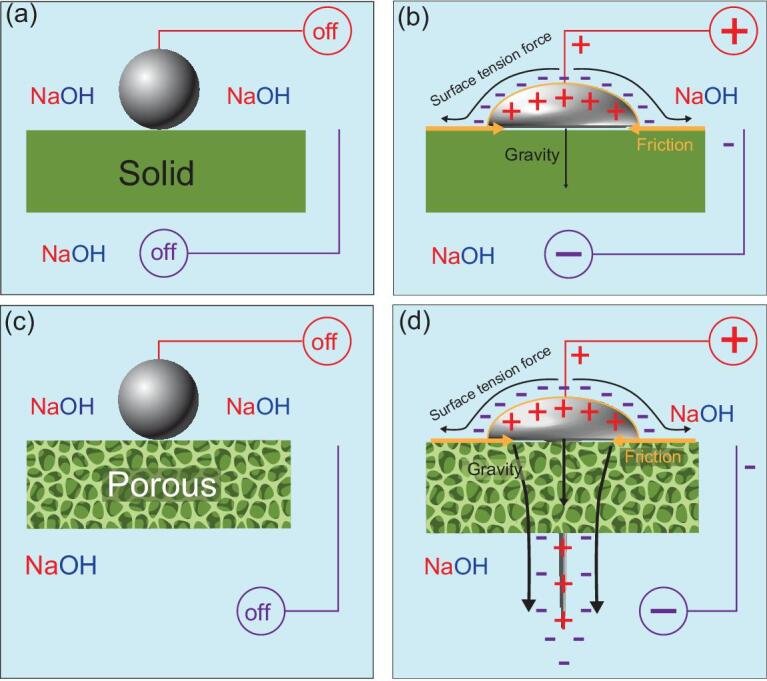
Schematic diagram of liquid metal spreading and penetration effect for a solid (a, b) and a porous material (c, d) with or without an applied voltage.

**Figure 5.**
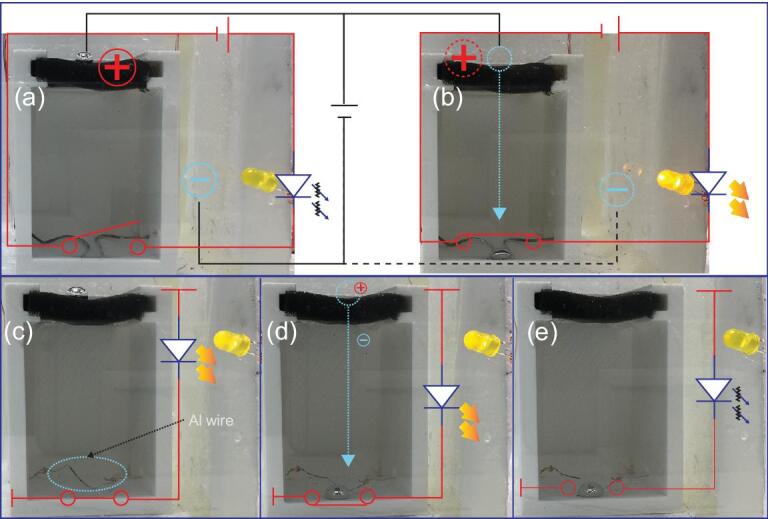
Potential applications: (a, b) healing a disconnected circuit in a sealed environment by penetrating a thick sponge. For clarity, the LED circuit is drawn in red, and gray lines describe the anode and cathode for the electrolyte: (a) the starting state of the process; (b) the final stage of the process, which is a connection in a previously open circuit. (c–e) Cutting off a closed circuit in a sealed environment, in which the liquid metal is first above a porous material, then penetrates through that porous material, reacts with the aluminum wire, and finally disconnects the circuit.

During the penetration process for all samples by cutting off the applied voltage, we found that once the voltage is turned off, the metal droplet stops penetration and reverses upwards back into the top surface of the porous material due to the recovery of the large surface tension of galinstan. Simultaneously, the thin metal threads extending down into the solution break up very quickly and reshape into spherical droplets at the bottom of the container.

For the mechanism of this fascinating phenomenon of liquid metal penetration of porous materials, it is well known that the flow of liquids is determined by two main factors: viscosity and surface tension. The almost zero surface tension that gives rise to the near-zero contact angle causes the liquid metal droplet to easily spread on any solid surface [9]. When no voltage is applied, the liquid metal droplet can remain in its spherical shape due to its high surface tension, as shown in Fig. 4a. After applying a voltage, the surface oxidation lowers the interfacial tension of the liquid metal droplet, which starts to spread. The influence of the external electric field (*V*) leads to a significant decrease in the surface tension gradient, which will produce a surface tension force (Fig. 4b) [26–28]. Moreover, the high density (∼6.359 g/cm^3^) of galinstan allows gravity to act on and contribute to its flow. Thus, the surface tension force generated from the external electric field and the gravity can simultaneously drive liquid metal to spread on the substrate by overcoming the friction, which is composed of both viscous and substrate friction. This process enables the continuous spreading of the liquid metal when in contact with a solid surface, regardless of the size of the contact area. As a result, the liquid metal can easily penetrate into any porous material. We assume a solid material with an appropriate porosity in which all voids are interconnected. When a droplet of liquid metal is placed on its surface, the liquid metal also remains stationary on the surface and will not penetrate through the solid due to its large surface tension (Fig. 4c). However, when a voltage is applied to the droplet, the droplet will start to diffuse into the solid and continues to flow into the interconnected voids until it finally penetrates through the solid, leaking out from the other side, under the surface tension force and the gravity (Fig. 4d). In principle, the liquid metal could flow through any porous materials provided that the external force generated from the electric field and gravity is larger than the fiction on the substrate and the viscous friction of the NaOH solution.

There are several potential applications of the liquid metal penetrating effect. Two possible applications are to heal and cut off electrical wiring using liquid metal in a sealed environment. Here, we demonstrate our experiments for the two possible applications. For the healing effect, disconnected copper wires are initially placed at the bottom of a container (Fig. 5a), while a light-emitting diode (LED) connected to the circuit is off. We then place a liquid galinstan droplet on the top surface of a sponge that is fixed 7 cm above the open circuit. After applying a voltage, the galinstan droplet penetrates through the sponge and flows down to the bottom. As a result, the two copper wires are physically connected by the liquid galinstan. The open circuit is then repaired, and the LED light is turned on (Fig. 5b).

We now demonstrate the cut-off effect. A closed electrical circuit is set up using an aluminum wire that is placed at the bottom of the container and the LED light is on. After the liquid metal penetrates through the sponge, the droplet drips down to the bottom and comes into direct contact with the aluminum wire while the LED light is still on. After about 2 min, however, the aluminum wire is cut by the liquid galinstan droplet due to the strong chemical reaction with gallium, and then the LED light turns off (Fig. 5c–e).

## CONCLUSION

In summary, we have discovered that liquid metal is capable of penetrating through any porous material at room temperature. We have demonstrated the penetration effect in sponges with different pore sizes and thicknesses as well as other porous materials such as tissue paper and different meshes. The key physics is that there is a giant reduction of the surface tension of the liquid metal to near-zero, induced by the applied voltage. The near-zero surface tension guarantees a contact angle of zero between the liquid metal and the solid surface. The near-zero surface tension drives the liquid metal to diffuse into the porous material and can be directed through the material using gravity. Our findings offer new opportunities for novel microfluidic applications and could promote further exploration for more exotic fluidic states of liquid metals.

## Supplementary Material

nwz168_Supplemental_Files
