# Supplementary material for: Voltage-induced penetration effect in liquid metals at room temperature
Source: Natl Sci Rev. 2019 Nov 5;7(2):366–72. doi: 10.1093/nsr/nwz168 (PMC8288959; doi:10.1093/nsr/nwz168)
Supplement: nwz168_Supplemental_Files [file nwz168_supplemental_files.zip › Supporting_Information-revised-1017.docx]

**Supplementary Information**

for

**Voltage induced penetration effect in liquid metals at room temperature**

Frank F. Yun^1^, Zhenwei Yu^1^, Yahua He^1^, Lei Jiang^2^, Zhao Wang^3^, Haoshuang Gu^3^, Xiaolin Wang^1,4,*^

1) Institute for Superconducting and Electronic Materials, Australian Institute for Innovative Materials, University of Wollongong, Wollongong, New South Wales 2500, Australia

2) Laboratory of Bioinspired Smart Interfacial Science, Technical Institute of Physics and Chemistry, Chinese Academy of Sciences, Beijing 100190, People's Republic of China

3) Faculty of Physics & Electronic Sciences, Hubei University, Wuhan, 430062 People's Republic of China

4) ARC Centre of Excellence in Future Low-Energy Electronics Technologies, University of Wollongong, Wollongong, New South Wales 2500, Australia

**METHODS**

Commercially available galinstan (62 wt % Ga, 22 wt % In, and 16 wt % Sn) alloys (99.99% purity, from Alfa Aesar) were used over lab-made samples of galinstan alloys to ensure maximum repeatability of results and to ensure availability of samples. Galinstan was picked for study specifically due to its very low melting temperature compared to other Ga alloy liquid metals so that its properties do not suffer from phase transitions caused by temperature fluctuations. Concentrations of 0.25-1 mol L^-1^ solutions were made using 99% solid NaOH dissolved in deionized water.

Several porous materials such as tissue paper, plastic, fiber, and metal meshes were used to demonstrate the persistent penetrating behavior of the phenomena A 3D printed plastic holder suspends the immersed plastic mesh in a 1 mol L^-1^ NaOH solution. A 150 µL galinstan droplet is placed on the top surface of the plastic mesh in the setup, as shown in Figure 1. The experiments were conducted under a voltage of 5 V DC, with the positive electrode contacted with the liquid galinstan, while the negative electrode being placed within the NaOH solution. The anode and cathode are two copper wires with a diameter of 0.5 mm. A video camera was placed in front of the container and recorded the whole progress of the experiment with 1080p horizontal resolution, at 33 frames-per-second (fps).

Three kinds of sponges with different average pore sizes are used. They are melamine foam with pore size of ~150 μm (Sponge A), Polyurethane foam with pore size of ~350 μm (Sponge B) and Polyurethane foam with pore size of ~550 μm (Sponge C). These sponges were cut into the thickness of 7.5 mm. Plastic holders were fabricated using a 3D printer with a 5×5 cm holding surface to hold onto the various porous surfaces at the height of 12 cm. These sponges were fixed in the plastic holder, then fully immersed into the NaOH solution with concentrations of 0.25-1 mol L^-1^. A 150 µL galinstan droplet is placed on the top surface of the sponges. The anode is contacted with the liquid galinstan, and the cathode can be put anywhere in the NaOH solution. The DC voltages were supplied from 0 to 20 V by a GW laboratory DC power supply (model GPS-1850). A video camera was placed in front of the container and recorded the whole progress of the experiment with 1080p horizontal resolution, at 33 frames-per-second (fps).

**SI Video. 1 |** The penetration effect for a sponge C (~550 μm) in 1 mol/L NaOH solution.

**SI Video. 2 |** The penetration effect for a 7.5 mm thickness sponge A (~150 μm) with 10 V applied voltage in 1 mol/L NaOH solution.

**SI Video. 3 |** The penetration effect for a 7.5 mm thickness sponge B (~350 μm) with 10 V applied voltage in 1 mol/L NaOH solution.

**SI Video. 4 |** The penetration effect for a 7.5 mm thickness sponge C (~550 μm) with 10 V applied voltage in 1 mol/L NaOH solution.


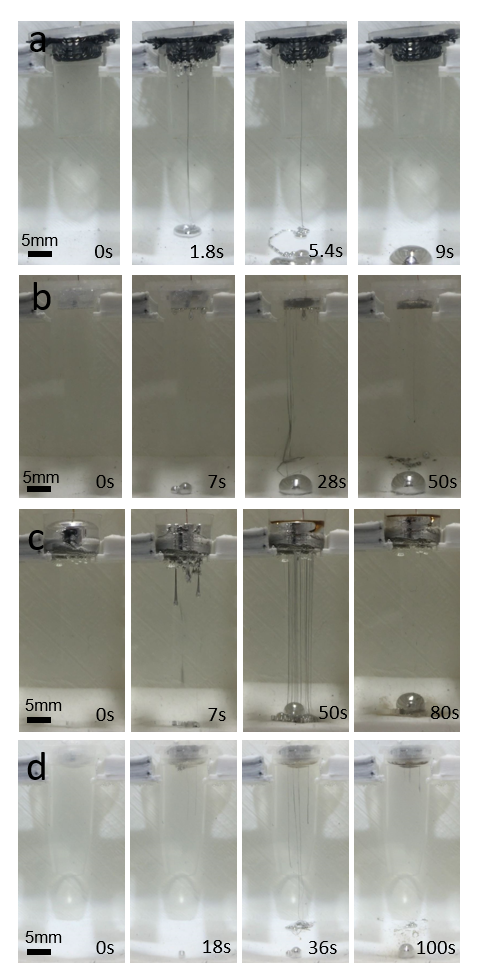


**Figure S1.** Snapshots of the penetration effect for (a) plastic mesh (pore size of 750 µm), (b) fabric mesh (pore size of ~ 280 mm) (c) metallic mesh (pore size of ~ 45 µm), and (d) wiper paper, with 5 V applied voltage in 1 mol/L NaOH solution.


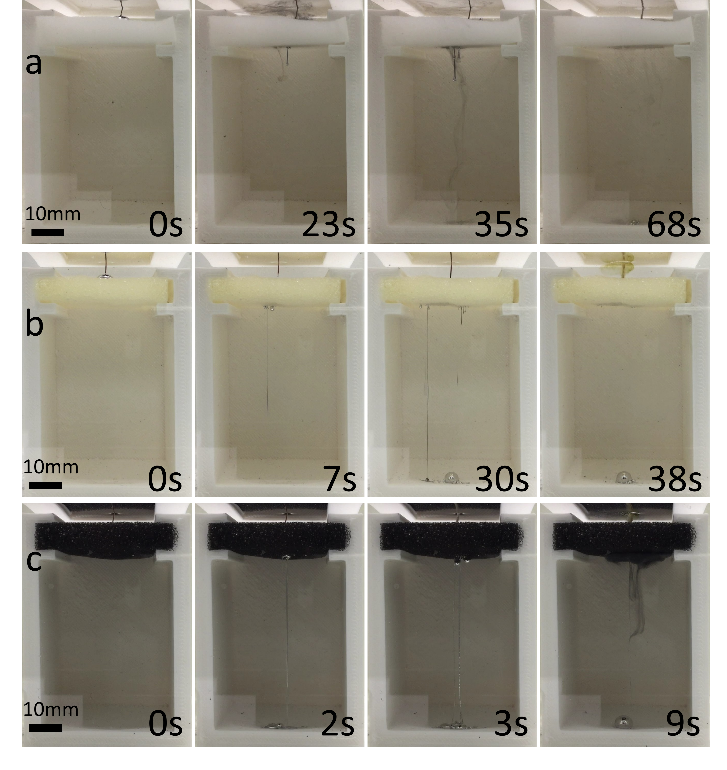


**Figure S2.** Snapshots of the penetration effect for (a) sponge A (~ 150 mm pore size), (b) sponge B (~ 350 mm pore size), and (c) sponge C (~ 550 mm pore size), each 7.5 mm in thickness with 10 V applied voltage in 1 mol/L NaOH solution.


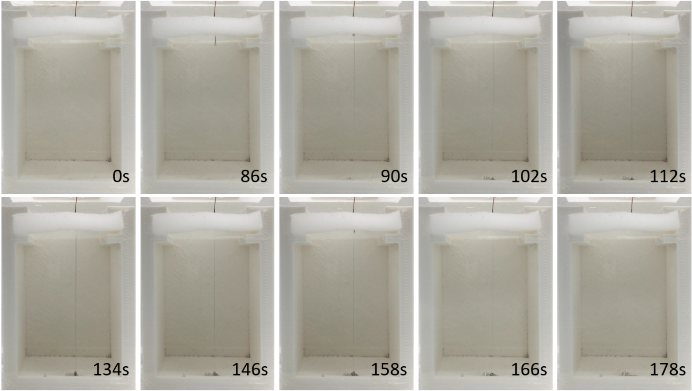


**Figure S3.** Snapshots of the penetration effect for a sponge A 7.5 mm in thickness with 2.5 V applied voltage in 0.25 mol/L NaOH solution.
